# Robotic vs. Standard Laparoscopic Technique – What is Better?

**DOI:** 10.3389/fsurg.2014.00015

**Published:** 2014-05-15

**Authors:** Ferdinand Köckerling

**Affiliations:** ^1^Department of Surgery and Center for Minimally Invasive Surgery, Vivantes Hospital Berlin, Academic Teaching Hospital of Charité Medical School, Berlin, Germany

**Keywords:** robotic surgery, laparoscopic surgery, meta-analysis, systematic review, oncological surgery

## Abstract

**Introduction:** Laparoscopic surgery is subject to certain limitations that can be a problem when performing complex minimally invasive operations. Robotic surgery was developed precisely to overcome such technical limitations. The question therefore arises whether robotic surgery leads to significantly better results compared with standard laparoscopic surgery.

**Methods:** Based on comparative systematic reviews and meta-analyses, this paper examines whether the robotic technique when used for abdominal and visceral surgery procedures confers advantages on the patient compared with the standard laparoscopic technique.

**Results:** Even for demanding visceral surgery procedures, the perioperative complication rate for robotic surgery is not higher than for open or laparoscopic surgical procedures. In cancer cases, the oncological accuracy of robotic resection for gastric, pancreatic, and rectal resection is seen to be adequate. Only the operating time is generally longer than for standard laparoscopic and open procedures. But, on the other hand, in some procedures blood loss is less, conversion rates are lower and hospital stay shorter.

**Conclusion:** To evaluate the future role of the robotic technique for visceral surgery, high-quality prospective randomized trials are urgently needed.

## Introduction

Laparoscopic surgery has certain limitations, such as two-dimensional imaging, restricted range of motion of the instruments, and poor ergonomic positioning of the surgeon (1). The robotic surgery system was introduced as a solution to minimize the shortcomings of laparoscopy (2). Improved visualization and greater dexterity are two major features of robotic-assisted laparoscopic surgery (3).

This emerging method provides undoubted technical advantages over conventional laparoscopy (4). Robotic systems have 3D imaging, tremor filter, and articulated instruments (5). With this advanced equipment, robotic surgery is superior to conventional laparoscopic surgery due to its significant improvements in visibility and manipulation (6, 7). Improvements in efficiency and usability of robotic systems are increasingly being explored (8).

Medical robotics is causing a paradigm shift in therapy (9). The most widespread surgical robot, the Intuitive Surgical’s da Vinci system, which has been discussed in over 4,000 peer-reviewed publications, was cleared by the United States’ Food and Drug Administration (FDA) for multiple categories of operations, and was used in 80% of radical prostatectomies performed in the USA in 2008, just 9 years after the system went on the market. Robotic prostatectomy is now the standard of care (5).

Robotics adoption in abdominal surgery has been slower than for other specialties due to the nature of abdominal surgery being highly varied throughout the abdomen and the advanced laparoscopic skill set possessed by minimally invasive surgeons (10).

But in visceral surgery, too, there has been a significant rise (*p* < 0.001) in the proportion of robotic operations performed in the USA from 0.8% in 2008 to 4.3% in 2009 (11). Based on a Nationwide Inpatients Sample Data Project, it was demonstrated that overall robotic surgery had a lower mortality rate (0.097%) than non-robotic surgical procedures per 10,000 procedures (laparoscopic 0.48%, open 0.92%; *p* < 0.001) (11). In all subgroups, robotic surgery had a significantly shorter hospital stay (4.9 days) than open surgery (6.1 days) and lower charges (median $ 30,540) than laparoscopic ($ 34,537) and open surgery ($ 46,704) (11). When the overall cost was considered, including length of stay, robotic surgery appeared to be cost-effective, although the cost of robotic surgery is generally considered a prohibitive factor (12).

## Materials and Methods

Based on comparative systematic reviews and meta-analyses, this study examines whether the robotic techniques when used for abdominal and visceral surgery procedures confers advantages on the patient compared with standard laparoscopic technique.

A systematic search of the available literature was performed in January 2014 of Medline, PubMed, Cochrane Library, and relevant journals and reference lists. The terms used for the search were: “robotic surgery” with 6,737 results, “robotic surgery and randomized controlled trials” with 133 results, and “robotic surgery and meta-analyses” with 15 results. Twenty-four articles were eligible for this review.

### Robotic vs. standard laparoscopic gastrectomy for cancer

A meta-analysis of seven studies with 1,967 patients compared robotic (*n* = 404) with open (*n* = 718) or laparoscopic (*n* = 845) gastrectomies (1). Robotic gastrectomy for gastric cancer reduces intraoperative blood loss and post-operative hospital stay compared with laparoscopic gastrectomy and open gastrectomy, but at the cost of a longer operating time. Robotic gastrectomy also provides an oncologically adequate lymphadenectomy.

In another meta-analysis, nine non-randomized comparative studies with 2,495 patients were included, of which 736 procedures were robotic and 1,759 were laparoscopic (13). Robotic gastrectomy was associated with lower intraoperative blood loss and shorter time to oral intake compared with laparoscopic gastrectomy. However, it was associated with a significantly longer operating time and shorter distal resection margin. In addition, there was no significant difference in the number of retrieved lymph nodes.

In another systematic review and meta-analysis, nine non-randomized observational clinical studies involving 7,200 patients satisfied the eligibility criteria (14). Robotic gastrectomy was associated with longer operating times than laparoscopic surgery and open gastrectomy (weighted mean difference 61.99 and 65.73 min, respectively; *p* ≤ 0.001). The number of retrieved lymph nodes and the resection margin length in robotic gastrectomy were comparable with those of laparoscopic and open gastrectomy. Estimated blood loss was significantly less in robotic gastrectomy than in open (*p* = 0.002), but not laparoscopic surgery. Mean hospital stay for robotic gastrectomy was similar to that for laparoscopic surgery (*p* = 0.14). In contrast, hospital stay was significantly shorter, by a mean of 2.18 days, for robotic gastrectomy compared with open surgery (*p* < 0.001). Post-operative complications were similar for all three operative approaches.

### Robotic vs. standard laparoscopic Roux-en-Y gastric bypass

A systematic review of robotic vs. laparoscopic Roux-en-Y gastric bypass identified 10 studies, which included results for 2,557 patients (15). The overall major and minor complications did not differ significantly between the robotic and laparoscopic group. The rates for anastomotic leak, bleeding, stricture, and reoperation did not differ significantly.

A systematic review and pooled analysis of robotic vs. laparoscopic Roux-en-Y gastric bypass in morbidly obese patients identified seven relevant studies of 1,686 patients (16). There was a significantly reduced incidence of anastomotic stricture in the robotic group (POR = 0.43; 95% CI: 0.19–0.98; *p* = 0.04). There was no significant difference between robotic and laparoscopic groups for anastomotic leak, post-operative complications, operative time, and length of hospital stay.

### Robotic vs. standard laparoscopic anti-reflux surgery

The University Health System Consortium collected in 115 academic institutions and their 271 affiliated hospitals a total of 12,079 patients with anti-reflux surgery. Of those, 2,168 were open fundoplications, 9,572 were standard laparoscopic, and 339 were robot-assisted laparoscopic fundoplications. There was no significant difference in mortality (0.1 vs. 0%; *p* = 0.5489), morbidity (4.0 vs. 5.6%; *p* = 0.1744), length of stay (2.8 ± 3.6 vs. 3.0 ± 3.5; *p* = 0.3242), and intensive care unit cases (8.4 vs. 11.5%; *p* = 0.051). The patients with standard laparoscopic fundoplication had a lower 30-day re-admission rate (1.8 vs. 3.6%; *p* = < 0.05) and cost (US $ 7,968 ± 6,969 vs. US $ 10,644 ± 6,041; *p* < 0.05) (17).

### Robotic vs. standard laparoscopic vs. open pancreatectomy

A meta-analysis of robotic-assisted pancreatectomy vs. laparoscopic and open pancreatectomy showed increased R0 resection rates and spleen preserving rates for the robotic approach in comparison with laparoscopic and open surgery. Moreover, robotic pancreatectomy can reduce estimated blood loss and duration of hospitalization more than open surgery (18).

### Robotic vs. standard laparoscopic colorectal procedures

A systematic review and meta-analysis of short-term outcome compared robotic rectal resection with laparoscopic resection for cancer (19). Eight non-randomized studies were identified, which included 854 patients in total, 344 (40.2%) in the robotic group and 510 (59.7%) in the laparoscopic group. Meta-analysis suggested that the conversion rate to open surgery in the robotic group was significantly lower than that with laparoscopic surgery (OR = 0.26; 95% CI: 0. 12–0.57; *p* = 0.0007). There were no significant differences in operation time, length of hospital stay, time to resume regular diet, post-operative morbidity and mortality, and the oncological accuracy of resection.

In a Nationwide Inpatient Sample 2009–2010, Halabi et al. (20) compared 3,568 robotic with 124,720 laparoscopic colorectal procedures. In the multivariate analysis, robotic surgery was associated with higher rates of post-operative bleeding in colonic cases (OR = 2.15; 95% CI: 1.27–3.65). Robotic colorectal surgery was similar to laparoscopic surgery with respect to length of hospital stay, morbidity, anastomotic leak, and ileus. Conversion to open surgery was significantly lower in robotic colonic and rectal procedures (0.41; 95% CI: 0.25–0.67) and (0.10; 95% CI: 0.06–0.16), respectively.

A meta-analysis of Yang et al. (21) included 16 studies comparing robotic-assisted laparoscopic surgery (*n* = 564) with standard laparoscopic surgery (*n* = 929) in patients with colorectal diseases including cancer and seven studies in rectal cancer (*n* = 300 RALS; *n* = 4.26 SLS). RALS was associated with lower estimated blood loss in colorectal diseases (*p* = 0.04) and rectal cancer (*p* = < 0.001) and lower rates of intraoperative conversion in colorectal diseases (*p* = 0.03) and rectal cancer (*p* = < 0.001). In contrast, operating time (*p* < 0.001) and total hospitalization cost (*p* = 0.06) were higher for RALS in the colorectal diseases group (21).

In another meta-analysis Memon et al. (22) found in comparison of 353 robotic-assisted laparoscopic rectal resections (low anterior resection, total mesorectal excision, coloanal anastomosis, and abdominoperineal resection) with 401 standard laparoscopic rectal resections a significantly lower conversion rate (*p* = 0.03). There was no difference in complications, hospital stay, distal resection margin, lymph node yield, or circumferential margin involvement (*p* = NS) (22).

Kim et al. (23) performed a systematic review about 69 studies (39 cases series, 29 comparative studies, and 1 randomized controlled trial) with 449 benign and 2,089 malignant colorectal cases. Most of the studies reported that robotic laparoscopic colorectal surgery showed less estimated blood loss, shorter length of hospital stay, lower complications and conversion rates, and comparable oncological outcomes and a larger operation time in comparison to standard laparoscopic colorectal surgery (23).

### Robotic vs. standard laparoscopic abdominal surgery

Maeso et al. (24) performed a systematic review and meta-analysis regarding the safety and efficacy of the da Vinci surgical system in abdominal surgery. The results found were subjected to meta-analysis whenever possible. Thirty-one studies, six of them randomized control trials, involving 2,166 patients that compared robotic surgery vs. laparoscopic surgery. The procedures undertaken were fundoplication, Heller myotomy, gastric bypass, gastrectomy, bariatric surgery, cholecystectomy, splenectomy, colorectal resection, and rectopexy. Robotic surgery was found to be associated with fewer Heller myotomy-related perforations, a more rapid intestinal recovery time after gastrectomy – and therefore a shorter hospital stay, a shorter hospital stay following cholecystectomy, longer colorectal resection surgery times, and a greater number of conversions to open surgery during gastric bypass.

## Conclusion

In summary it can be stated that, when all influence factors are taken into account, robotic surgery need not necessarily be more expensive than open and laparoscopic surgery. Even for demanding visceral surgery procedures, the perioperative complication rate for robotic surgery is not higher than for open or laparoscopic surgical procedures. In cancer cases, the oncological accuracy of robotic resection for gastric, pancreatic, and rectal resection is seen to be adequate. Only the operating time is generally longer than for standard laparoscopic and open procedures. But, on the other hand, in some procedures blood loss is less, conversion rates are lower, and hospital stay shorter.

To evaluate the future role of the robotic technique for visceral surgery, high-quality prospective randomized trials are urgently needed. To that effect, surgeons should definitely have mastered the learning curve. But already the existing evidence indicates that robotic surgery will have a permanent future role in visceral surgery. Therefore visceral surgeons should actively contribute to further development of robotic surgery and initiate high-quality comparative studies in this area.

## Conflict of Interest Statement

The author declares that the research was conducted in the absence of any commercial or financial relationships that could be construed as a potential conflict of interest.
